# Mechanisms of Key Innate Immune Cells in Early- and Late-Onset Preeclampsia

**DOI:** 10.3389/fimmu.2020.01864

**Published:** 2020-08-18

**Authors:** Ingrid Aneman, Dillan Pienaar, Sonja Suvakov, Tatjana P. Simic, Vesna D. Garovic, Lana McClements

**Affiliations:** ^1^Faculty of Science, School of Life Sciences, University of Technology Sydney, Sydney, NSW, Australia; ^2^Division of Nephrology and Hypertension, Department of Internal Medicine, Mayo Clinic, Rochester, MN, United States; ^3^Faculty of Medicine, Institute of Medical and Clinical Biochemistry, University of Belgrade, Belgrade, Serbia; ^4^Department of Medical Sciences, Serbian Academy of Sciences and Arts, Belgrade, Serbia

**Keywords:** immune cells, pregnancy, late-onset preeclampsia, early-onset preeclampsia, preeclampsia, inflammation, innate immunity

## Abstract

Preeclampsia is a complex cardiovascular disorder of pregnancy with underlying multifactorial pathogeneses; however, its etiology is not fully understood. It is characterized by the new onset of maternal hypertension after 20 weeks of gestation, accompanied by proteinuria, maternal organ damage, and/or uteroplacental dysfunction. Preeclampsia can be subdivided into early- and late-onset phenotypes (EOPE and LOPE), diagnosed before 34 weeks or from 34 weeks of gestation, respectively. Impaired placental development in early pregnancy and subsequent growth restriction is often associated with EOPE, while LOPE is associated with maternal endothelial dysfunction. The innate immune system plays an essential role in normal progression of physiological pregnancy and fetal development. However, inappropriate or excessive activation of this system can lead to placental dysfunction or poor maternal vascular adaptation and contribute to the development of preeclampsia. This review aims to comprehensively outline the mechanisms of key innate immune cells including macrophages, neutrophils, natural killer (NK) cells, and innate B1 cells, in normal physiological pregnancy, EOPE and LOPE. The roles of the complement system, syncytiotrophoblast extracellular vesicles and mesenchymal stem cells (MSCs) are also discussed in the context of innate immune system regulation and preeclampsia. The outlined molecular mechanisms, which represent potential therapeutic targets, and associated emerging treatments, are evaluated as treatments for preeclampsia. Therefore, by addressing the current understanding of innate immunity in the pathogenesis of EOPE and LOPE, this review will contribute to the body of research that could lead to the development of better diagnosis, prevention, and treatment strategies. Importantly, it will delineate the differences in the mechanisms of the innate immune system in two different types of preeclampsia, which is necessary for a more personalized approach to the monitoring and treatment of affected women.

## Introduction

Preeclampsia accounts for over 70,000 maternal and 500,000 fetal/neonatal deaths annually, with maternal deaths being highest in developing countries (1, 2). The exact etiology of preeclampsia is unknown, however, endothelial dysfunction, inappropriate angiogenesis, inadequate trophoblast invasion and spiral uterine artery remodeling, have all been identified as key contributors (3–6). Adequate remodeling of spiral uterine arteries into dilated, elastic, and low-resistance blood vessels enables unlimited supplies of oxygen and nutrients to the fetus. This requires appropriate invasion by extravillous trophoblasts and replacement of maternal endothelial cells (7). Inappropriate activation of the innate immune system and subsequent inflammation, however, can lead to placental dysfunction or poor maternal vascular adaptation and contribute to the development of preeclampsia (8). In this review, we will outline mechanisms of key innate immune cells implicated in the development of preeclampsia and differentiate how these mechanisms are affected in two phenotypes of preeclampsia, early-onset preeclampsia (EOPE) and late-onset preeclampsia (LOPE). The 2018 recommendations from The International Society for the Study of Hypertension in Pregnancy (ISSHP) define preeclampsia as *de-novo* hypertension (systolic blood pressure > 140 mmHg and diastolic blood pressure > 90 mmHg) after 20 weeks of gestation, accompanied by one or more of the following features: proteinuria (>300 mg/day), maternal organ dysfunction (including hepatic, renal, neurological), or hematological involvement such as thrombocytopenia, and/or uteroplacental dysfunction, such as fetal growth restriction and/or abnormal Doppler ultrasound findings of uteroplacental blood flow (1, 2, 9). Preeclampsia with severe features is defined as cases with blood pressure values ≥160/110 mmHg, accompanied by significant proteinuria (≥300 mg of protein/day), or pulmonary edema, cerebrovascular and/or liver function deterioration or thrombocytopenia (10). EOPE is diagnosed before 34 weeks of gestation whereas LOPE is diagnosed from 34 weeks of gestation (2).

## Incidence and Treatment of Preeclampsia

A systematic review of the incidence of hypertensive disorders of pregnancy, including 39 million women from 40 countries, found that preeclampsia affects ~4.6% of all deliveries globally (11). Another review reported that preeclampsia complicates 2 to 8% of pregnancies (12). The reasons for differences in the incidence of preeclampsia among different countries, regions or hospitals include inconsistencies in the diagnostic criteria, difficulty in diagnosing preeclampsia, as well as differences in maternal age and nulliparity, access to prenatal care and education, and regional prevalence of other risk factors (2, 11, 13). Women who have chronic hypertension, autoimmune disorders, kidney disease, pre-gestational diabetes, maternal body mass index (BMI) > 30 kg/m^2^ and a family or personal history of preeclampsia, are at higher risk of developing preeclampsia; older age (>40 years) is also associated with increased risk of preeclampsia (1). Treatment of preeclampsia can be divided into expectant care and interventionist care (14). Expectant care involves a balance of stabilizing the mother's condition and delaying delivery as far as the maternal condition allows, to reduce the mortality and morbidity associated with premature birth. Interventionist care involves early delivery to minimize serious maternal and fetal complications. Expectant care provides relief from symptoms, such as reducing blood pressure with antihypertensive therapy and the use of magnesium sulfate as anticonvulsant therapy (2, 15, 16). Evidence suggests that there is no clear difference between an expectant or interventionist care approach for preeclampsia with severe features (14). Without a clear contraindication, delaying delivery for as long as possible can improve outcomes for the fetus (17). Studies investigating the prophylactic use of aspirin in high-risk pregnancies have reported conflicting findings (9, 18). A meta-analysis including 18,907 women concluded that when taken before 16 weeks of gestation at a daily dose of ≥100 mg, aspirin could reduce the risk of preterm preeclampsia diagnosed before 37 weeks of gestation (19). As such, high-risk patients must be identified early in pregnancy for any beneficial effects to be observed (16). Calcium supplementation for women with low calcium diets may lead to a reduction in the severity of symptoms associated with preeclampsia and minimize the risk of preterm birth (2, 18). In the case of diabetic pregnancies, women who were given metformin with and without insulin treatment had a lower incidence of preeclampsia (20).

## Similarities and Differences Between Early-Onset and Late-Onset Preeclampsia

Gestational age has been identified as the most important clinical variable in predicting both maternal and perinatal outcomes (21). This led to stratification of preeclampsia into two phenotypes, EOPE and LOPE (1, 2, 22). LOPE accounts for the majority of preeclampsia cases, comprising ~80 to 95% of all preeclampsia cases worldwide (23). EOPE, although less common, is associated with higher rates of neonatal mortality and a greater degree of maternal morbidity compared to LOPE (3). As a result, EOPE has attracted greater interest and more studies have focused on elucidating the mechanisms underlying this disease phenotype (23), leading to implementation of preventative treatments (e.g., aspirin) and predictive biomarkers more suited for EOPE than LOPE. LOPE, nevertheless, is also a serious condition, associated with a high prevalence of eclampsia and HELLP (hemolysis, elevated liver enzymes, and low platelets) syndrome, which are two life-threatening complications (24). Further studies are needed to address this gap in research. Preeclampsia has been described as a two-stage disease, with initial deficient remodeling of the uterine spiral arteries leading to a stage of maternal systemic inflammation and vascular dysfunction (25). This model is more representative of EOPE. Impaired placental development in early pregnancy and subsequent growth restriction is often associated with EOPE, while LOPE is likely associated with maternal endothelial dysfunction (26). Both phenotypes exhibit an increased inflammatory response that leads to adverse maternal and fetal complications. Syncytiotrophoblast stress and placental hypoxia are implicated as the main cause of excessive systemic vascular inflammation. In EOPE, this is triggered by dysfunctional perfusion of the placenta. In the case of LOPE, syncytiotrophoblast stress likely occurs as a result of compression of placental terminal villi, as the placenta outgrows the space within the uterine cavity, which can also lead to uteroplacental malperfusion (24, 27).

Timely detection of preeclampsia is complicated by the fact that the disease is usually asymptomatic in its early stages (2). Close antenatal monitoring, especially during the third trimester, can be crucial in preventing maternal and fetal complications. Detection using angiogenesis-related biomarkers such as the ratio of soluble fms-like tyrosine kinase-1 (sFlt-1) and placental growth factor (PIGF), as well as Doppler ultrasound assessment, can be useful in detecting EOPE, and to a lesser extent LOPE. Recently, other angiogenesis-related biomarkers, FKBPL and CD44, were also implicated in prediction and diagnosis of preeclampsia, particularly LOPE (28). Further research is needed to elucidate the pathogenic mechanisms and develop diagnostic biomarkers for LOPE early in pregnancy, allowing interventions to begin before clinical features manifest.

## Complications Associated With Preeclampsia

Women with a history of preeclampsia, in addition to short-term complications, have a higher risk of subsequent cardiovascular and metabolic disorders, especially following EOPE (29). A meta-analysis including datasets from 3,488,160 women found that women with previous preeclampsia were twice as likely to develop ischemic heart disease compared to normotensive pregnancies (30). It is not clear whether this increased risk of subsequent cardiovascular disease is caused by underlying maternal risk factors, which are exacerbated by preeclampsia, or if this increased risk is a consequence of preeclampsia (2). Potential overlapping mechanisms between preeclampsia and cardiovascular disease including hypertension and/or heart failure with preserved ejection fraction were recently identified using a bioinformatics “*in silico*” approach (31, 32).

Untreated preeclampsia, regardless of the phenotype, can result in severe complications including liver rupture, cerebral hemorrhage, myocardial infarction, stroke, acute respiratory distress syndrome, pulmonary edema, kidney failure, and abruptio placentae (1, 9, 16). Delivery of the baby, even if it is preterm, minimizes the risk of developing these maternal and fetal complications, including fetal growth restriction and fetal loss. Premature birth, nevertheless, is also associated with a number of neonatal complications such as respiratory distress syndrome, intraventricular hemorrhage, and necrotizing enterocolitis (14). While there are a multitude of factors that contribute to the pathogenesis and onset of preeclampsia, in recent years, it has been highlighted that an overactive maternal immune system can play a critical role in preeclampsia development.

## Innate Immune System in Healthy Pregnancy and Preeclampsia

**Figure 1:**
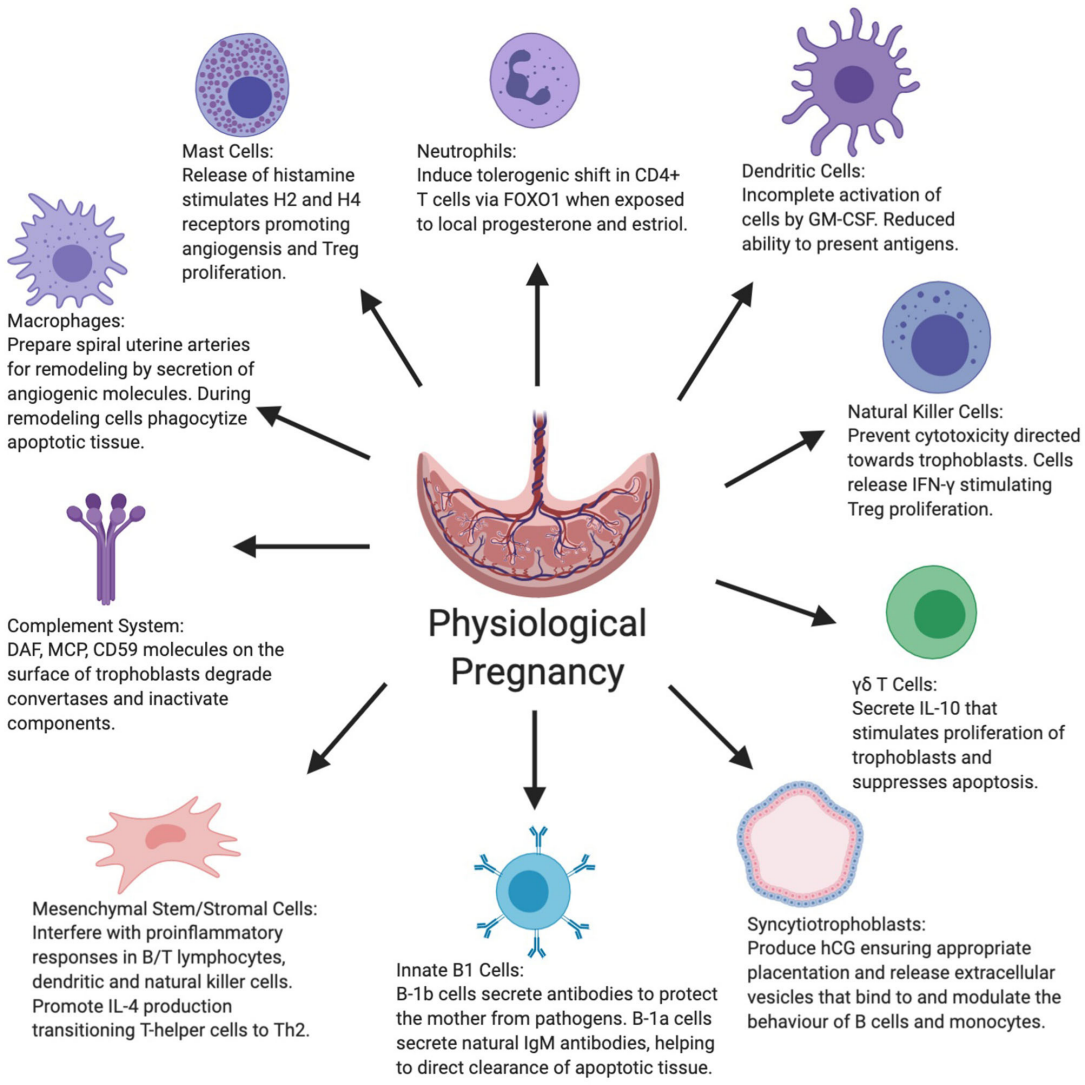
Figure summarizes the various roles displayed by the innate and other key immune system cells as well as syncytiotrophoblasts and mesenchymal stem cells in normal physiological pregnancy. CD59+, Cluster of differentiation 59+; DAF, Decay-accelerating factor; FOXO1, Forkhead box protein-1; GM-CSF, Granulocyte-macrophage colony-stimulating factor; hCG, Human chorionic gonadotropin; IFN-γ, Interferon-gamma; IL-4, Interleukin-4; IL-10, Interleukin-10; MCP, Membrane cofactor protein.

**Figure 2:**
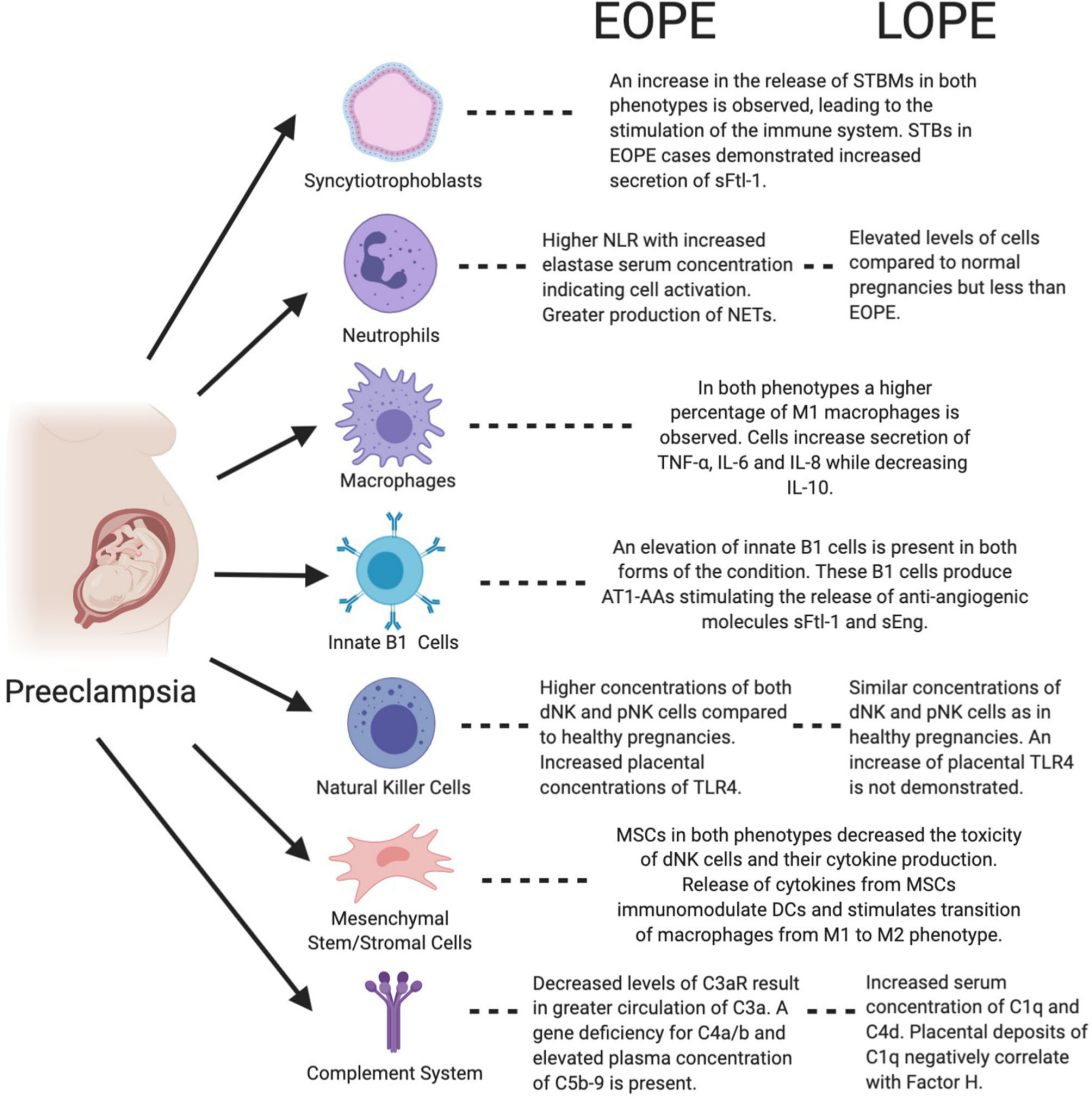
Figure highlights the functions of both immune and placental cells in the context of preeclampsia and both of its phenotypes, EOPE and LOPE. AT1-AA, Angiotensin II type 1 receptor agonistic autoantibody; DC, Dendritic cell; dNK, Decidual natural killer; IL-6, Interleukin-6; IL-8, Interleukin-8; IL-10, Interleukin-10; MSC, Mesenchymal stem/stromal cell; NET, Neutrophil extracellular trap; pNK, Peripheral natural killer; NLR, Neutrophil to lymphocyte ratio; sEng, Soluble Endoglin; sFtl-1, Soluble fms-like tyrosine kinase-1; STB, Syncytiotrophoblast; STBM, syncytiotrophoblast micro-particles; TLR4, Toll-like receptor 4; TNF-α, Tumor necrosis factor-α.

The maternal innate immune system throughout the entire gestation period plays an important role in ensuring protection from pathogens, while concurrently inducing tolerance to the semi-allogeneic developing fetus and placental development. As outlined in Figure 1, this is achieved through a delicate balance of various cell functions and interactions between the innate immune system cells and other placental/uterine cells in a timely manner (33, 34). Unfortunately, this is not always the case and due to various factors, the aforementioned balance is disrupted by maladaptation of certain immune cells during gestation, which is demonstrated in Figure 2. In physiological pregnancies, decidual macrophages found in proximity to spiral uterine arteries help prepare these for remodeling via secretion of angiogenic molecules (35–37). Macrophages also phagocytize apoptotic cells during tissue remodeling, preventing the release of self-antigens or paternal alloantigens, which could trigger a maternal immunological response (38). There are two phenotypes of macrophages, M1 or classically activated macrophages, and M2 or alternatively activated macrophages. M1 macrophages are involved in phagocytosis, and are micro-biocidal and pro-inflammatory. M2 macrophages are immunomodulatory and responsible for inducing maternal tolerance, resolving inflammation, and are involved in tissue remodeling and cell proliferation (39, 40). Therefore, in normal physiological pregnancy, macrophages favor the M2 phenotype, whereas in preeclampsia, this balance is shifted toward the M1 phenotype (41). M1 cells secrete soluble fms-like tyrosine kinase-1 (sFlt-1), an anti-angiogenic molecule that is associated with impaired angiogenesis in preeclampsia (42). Consequently, the transition of macrophage phenotype from M2 to M1 is indicative of a pro-inflammatory response as observed in preeclampsia.

## Immunomodulation by Innate Immune Cells

Innate immune cells, while assisting in the initial stages of pregnancy, also exhibit immunomodulatory characteristics targeted at immunological responses toward the fetus. Mast cells have demonstrated such characteristics through the release of histamine and stimulation of the G protein-coupled receptors (H_1−4_R) (43). Activation of the H_4_R appears to lead to proliferation of regulatory T cells (Tregs), and H_2_R support angiogenesis at the fetal-maternal interface (43, 44). Tregs subsequently act to prevent lymphocytes from attacking the fetus. There is limited evidence regarding the behavior of mast cells during preeclampsia and their contribution to its onset. There are some reports that they accumulate at higher density adjacent to spiral uterine arteries during preeclampsia and undergo intensive degranulation, leading to a release of large concentrations of histamines (45). The high histamine concentrations in the circulation stimulate pro-inflammatory responses from both the innate and adaptive immune system, leading to the secretion of pro-inflammatory cytokines and molecules, contributing to the increase in blood pressure, typical of preeclampsia (46).

## The Role of Neutrophils in Maternal Tolerance During Pregnancy

Neutrophils, also as part of a normal physiological pregnancy, are recruited to the developing placenta via the chemokine, IL-8 (47). Following the recruitment of circulating neutrophils to the placenta and under the influence of progesterone and estriol, CD4^+^ T cells undergo immunomodulation via transfer of the forkhead box protein 1 (FOXO1) from neutrophils (48). These neutrophil-induced T (niT) cells, in addition to establishing maternal tolerance of the fetus, secrete IL-10, IL-17, and vascular endothelial growth factor (VEGF) promoting angiogenic processes (48). Also in normal physiological pregnancy, the natural cytotoxicity receptors (NCRs) on maternal decidual natural killer (NK) cells are inactivated to ensure maternal tolerance. This is not the case in preeclampsia due to abnormalities in the NCR structure, which increases their synthesis and expression on NK cells, thus affecting maternal tolerance to the fetus (49). Overactivation of NCRs on NK cells leads to discharge of pro-inflammatory cytokines, thus contributing to weakened immunological tolerance potentially endangering the fetus (50).

## Inactivation of Innate Immune Cells in Pregnancy

Whereas the aforementioned cells either assist in required gestational processes or exhibit immunoregulatory roles, other innate immune cells become partially or fully inactivated during pregnancy. NK cells within the uterus and decidua, for example, maintain their immunological function against infections without compromising the fetus. This is achieved by inhibiting NK cell-mediated cytotoxicity toward trophoblasts by interfering with their degranulation process (51). NK cells additionally release IFN-γ, which stimulates decidual CD14^+^ myelomonocytic cells to induce Treg proliferation via transforming growth factor-beta (TGF-β) (52). The incomplete activation of DCs by granulocyte-macrophage colony-stimulating factor (GM-CSF) similarly leads to a reduction in the capacity of these cells to adequately present antigens (53). This effect consequently limits the development and activation of T cells, increasing maternal tolerance toward the fetus (54). DCs located proximally to the placenta remain inactivated or immature during normal pregnancy; however, these cells become inappropriately stimulated during preeclampsia. Although GM-CSF initially acts as a regulator of DCs, in higher concentrations, GM-CSF along with lnc-DC (long non-coding RNA expressed in DCs), induces DC maturation (55, 56). Once mature, DCs can more efficiently present antigens leading to an increase in the proliferation of Th1/Th17 cells (56, 57). The Th1/Th17 cells consequently stimulate pro-inflammatory responses, which significantly reduce maternal tolerance.

## B1 Cell Activation in Healthy Pregnancy and Preeclampsia

B1 cells, despite originating from the lymphoid group of immune cells, also belong to the innate immune system and are important in the initiation and maintenance of a healthy pregnancy. The B1 cell population is subdivided into two groups, B-1a and B-1b, each exhibiting unique roles throughout gestation (58). B-1b cells produce typical antibodies in response to antigen identification, providing protection against invading pathogens (58). On the other hand, B-1a cells secrete “natural IgM antibodies” with low-affinity, poly-reactivity, and self-reactivity, regardless of antigenic stimuli (58, 59). These natural antibodies assist in the clearance of apoptotic tissue cell bodies, affecting the immune response during tissue remodeling (59). B1 innate cells, similar to other immune cells, are irregularly activated in preeclampsia. Notably, B-1a cells are stimulated to produce angiotensin II type 1 receptor agonistic autoantibodies (AT1-AA) in preeclampsia, which does not occur in normal physiological pregnancies (60). These antibodies, as the name suggests, act as agonists and induce signaling pathways, leading to the vasoconstriction of blood vessels and the secretion of aldosterone, which stimulates the renin-angiotensin system and increases blood pressure (61).

## Influence of the Complement System

The capacity of the complement system during healthy physiological pregnancy is also modulated to prevent its activation, which could endanger the fetus. Regulatory proteins including decay-accelerating factor (DAF), membrane cofactor protein (MCP), and CD59, found on the surface membrane of trophoblasts, help to degrade convertases within the complement system inducing components of this system into their inactive forms by way of cleavage (62). Due to the widespread dysregulation of the maternal immune system during preeclampsia, the complement system becomes overstimulated as part of a compensatory mechanism. Over-activation of both the classical and lectin complement pathways leads to greater terminal activation, causing inflammation and the recruitment of large numbers of phagocytes to the origin of the stimuli (63). This subsequently contributes to the onset of maternal hypertension and organ damage (63).

## Syncytiotrophoblast Cells and Their Secreted Extracellular Vesicles

The blastocyst, in anticipation of implantation, initiates secretion of human chorionic gonadotropin (hCG) before its synthesis is superseded by syncytiotrophoblast cells (STBs) (7). The continued secretion of hCG by STBs ensures appropriate invasion of trophoblast cells into the endometrium. Upon shedding of the STB layer, extracellular vesicles (EVs) are released from apoptotic STBs, which bind to monocytes and B cells (64). EVs encompass three main vesicle types: exosomes, micro-particles/micro-vesicles and apoptotic bodies (65–67). These EVs, upon binding, induce a shift in the cytokine secretion profile of the neighboring cells, causing the release of anti-inflammatory cytokines (64). On the other hand, in preeclampsia when placental ischemia and hypoxia are present, a greater number of the STB cells undergo apoptosis (68). There is subsequently an increase in secreted EVs into the maternal circulation, overwhelming the body's ability to adequately scavenge and clear them effectively (68, 69). These vesicles then act as antigenic stimuli for components of the immune system leading to unintended endothelial injury, inflammation, and hyper-coagulation (68).

## Immunomodulation by Mesenchymal Stem/Stromal Cells

Emerging evidence suggest that mesenchymal stem/stromal cells (MSCs) also have important immunomodulatory roles in pregnancy. Facilitated by paracrine signaling, MSCs target B/T lymphocytes, DCs, and NK cells and interfere with their pro-inflammatory responses. Simultaneously, MSCs stimulate the shift of T-helper cells from a Th1 to Th2 phenotype by promoting IL-4 production and inhibiting IFN-γ production, thus heightening maternal immunological tolerance of the fetus. Furthermore, MSCs have also been shown to have a key pro-angiogenic role in pregnancy (70). In preeclampsia, the function of MSCs is likely impaired due to exposure to increased numbers of reactive oxygen species (ROS) (70, 71). The presence of aldehyde dehydrogenases (ALDH) during normal pregnancy assists in detoxification from ROS, thus providing a degree of protection against oxidative damage (71). However, due to an unknown mechanism, levels of ALDH in preeclampsia are decreased, exposing the MSCs to oxidative stress with resulting damage and reduced functional ability to modulate other immune cells (71).

In summary, immune cells are critical in the progression of normal physiological pregnancy, both in terms of maternal tolerance and placental development; the roles of key innate and other immunogenic cells in normal physiological pregnancy are depicted in Figure 1. The vast majority of immune cells and other associated placental/uterine cells become over-activated or dysregulated in preeclampsia, contributing to the overall symptoms and features of the condition, including hypertension and organ damage.

## Macrophage Phenotype Plasticity in Preeclampsia

Macrophages can alter their phenotypic profiles in response to a variety of environmental factors (39). M1 macrophages develop in response to exposure to Th1 cytokines such as IFN-γ, tumor necrosis factor (TNF)-α and lipopolysaccharide (LPS) (72, 73). The development of M2 macrophages is favored in the presence of TGF-β, IL-4, IL-10, and IL-13. Inflammatory cytokines, TNF-α, IL-6, and IL-8, are significantly increased in preeclampsia, and IL-10 is significantly decreased compared to normal pregnancy, therefore promoting the M1 phenotype (74, 75). Decidual macrophages comprise 20% of the immune cells present within the placenta (76). Placental decidual macrophages in normal pregnancy are mainly of the M2 subset and can be found near spiral uterine arteries (40). They have an important role in preparing spiral uterine arteries for remodeling by trophoblasts, as well as phagocytozing pro-inflammatory substances formed during the process of remodeling. The predominance of the M1 decidual macrophage phenotype is conducive to the release of substances such as TNF- α, IFN-y and nitric oxide (NO), which inhibit trophoblast invasion and spiral uterine artery remodeling (39, 72, 76). Various studies have reported increased levels of decidual macrophages in preeclampsia (55, 77, 78). Other studies have reported a reduction in macrophages in the placental decidua in preeclampsia, possibly due to reduced monocyte migration to the decidua or lack of differentiation into macrophages (79, 80). Chitinase-3-like protein 1 (CHI3L1), also known as YKL-40, is indicative of the number of macrophages, and it has been shown to be present in significantly lower levels in women who developed EOPE compared to normal pregnancy (81). These conflicting findings may be a result of different macrophage cell markers, methods employed, use of tissue samples from different preeclampsia phenotypes and different sections of the placenta being studied, considering that decidual macrophages reside predominantly around spiral uterine arteries (82).

## Markers of Systemic Inflammation: Neutrophil Extracellular Traps and the Neutrophil-Lymphocyte Ratio

Neutrophils are likely the main class of leukocytes that cause the majority of vascular cell dysfunction in women with preeclampsia (83). Neutrophil activation may occur from exposure to oxidized lipids secreted by the placenta as a consequence of placental damage. Activated neutrophils infiltrate the maternal systemic vasculature and release substances such as ROS, TNF-α and myeloperoxidase (MPO), causing endothelial dysfunction (74, 83). MPO has been associated with hypertension, and elevation in TNF-α is recognized as a driving inflammatory mechanisms to preeclampsia (84, 85).

Neutrophil numbers in the maternal systemic circulation and within the decidua steadily increase in pregnancy throughout gestation, yet this increase is further amplified in preeclampsia (4, 74, 86–88). Elevation in neutrophil count has been noted in both EOPE and LOPE compared to normal pregnancy, with a greater elevation present in EOPE *(*26). This surge in neutrophils may be an adverse reaction to the interaction between the maternal immune system and micro-debris originating from the placenta (89). Although the number of neutrophil granulocytes increases, the phagocytic function of these cells reportedly decreases in pregnancy, particularly in preeclampsia (88). Plasma elastase, a marker of neutrophil activation, has been noted to be elevated in preeclampsia when compared to normal pregnancy (90). A small sample cohort study reported a significant increase in plasma elastase in EOPE compared to normotensive controls (3). Neutrophil extracellular traps (NETs) have been found in the intervillous spaces of placentae in women with preeclampsia (25). The formation of these web-like chromatin structures is induced by STB microparticles (STBMs) released from the placenta and ROS (91). Neutrophils, in addition to causing inflammation, represent the first wave of leukocytes responding to inflammation (5, 83). NETs are abundant within sites of inflammation causing endothelial damage as demonstrated in cases of sepsis, and may also cause damage to villous trophoblast cells in preeclampsia (92, 93). The presence of NETs in the maternal circulation during pregnancy can contribute to thrombotic events, inflammation, and ultimately, fetal death (94). The neutrophil-to-lymphocyte ratio (NLR), is a measure of systemic inflammation, and has demonstrated prognostic value in several cardiovascular diseases, including preeclampsia (4). Both normal pregnancy and preeclampsia present with an increased NLR compared to non-pregnant controls (95). Several studies, however, have reported the NLR to be significantly higher in women with preeclampsia compared to normotensive controls (6, 16, 96, 97). A retrospective case-control study conducted with 186 patients found the NLR to be highest in EOPE, and LOPE demonstrating higher NLRs compared to normotensive controls (26). A change in the NLR can be noted at 16–18 weeks of gestation, and thus has a potential as an inexpensive biomarker for the early detection, monitoring and prompt intervention particularly for EOPE (97).

## Natural Killer Cells in Preeclampsia

As discussed previously, in normal pregnancy the appropriate remodeling of spiral uterine arteries into low resistance and high capacity vessels coordinated with appropriate trophoblast invasion is pivotal. When these two processes are not well-coordinated, the consequent insufficient blood flow leads to a series of events, which ultimately result in the development of preeclampsia. This suggests that interactions at the maternal-fetal interface in early gestation are important for determining the course of pregnancy. In order to preserve an adequate immuno-tolerant environment, DCs and T-lymphocytes have limited access to the decidua during pregnancy (98). NK cells, in fact, represent 70% of the immune cells in the decidua (99, 100). These decidual NK (dNK) cells are a distinct population from peripheral NK (pNK), both phenotypically and functionally. Unlike pNK, the dNK subpopulation has a CD56^+^/CD16^−^ phenotype (101) and demonstrates a lower cytotoxic potential and higher cytokine secretory profile (102). Decidual NK cells, by secreting VEGF and PlGF, stimulate spiral uterine artery remodeling, a process crucial for successful establishment of the placenta and the feto-maternal interface in pregnancy (103, 104). The lack of dNK cells has been shown to lead to lower fertility and higher fetal resorption (105). NK cells, on the other hand, are recruited by the innate immune system in response to inadequate trophoblast invasion or insufficient spiral uterine artery remodeling, processes observed in preeclampsia. There are some inconsistencies among studies, however, with respect to the number of these cells present in preeclampsia compared to normotensive pregnancy. While some studies have reported significantly lower numbers of NK CD56^+^ cells within the decidua in preeclampsia (77, 106), other reports have indicated the opposite trend (107, 108). The heterogeneity among studies and the differences in patient characteristics offer possible explanations for these discrepancies. A recent study demonstrated that the increases in both dNK and pNK cells were higher in EOPE compared to LOPE (108).

## Toll-Like Receptors in Preeclampsia

Toll-like receptors (TLRs) represent a family of transmembrane signaling receptors found on all innate immune cells. Ten different TLRs have been identified in humans based on their cellular localization and respective ligands (109). All 10 TLRs activate nuclear factor κB- (NF-κB) dependent and NF-κB-independent pathways to generate cytokines and chemokines (109). TLR expressions vary throughout pregnancy. Trophoblast expression of TLRs changes throughout gestation, with TLR2-4 being highly expressed during the first trimester and TLR1-10 in the third trimester (110–114). TLRs activate inflammatory responses by recognizing damage-associated molecular patterns (DAMPs) released following tissue injury, as well as pathogen-associated molecular patterns (PAMPs) specific to microbial components (115–117). Continuous signaling from DAMPs due to persistent cell death and remodeling of spiral uterine arteries leads to over-activation of TLRs. Excessive TLR activity may contribute to the pro-inflammatory effects and hypertension observed in preeclampsia. Studies report that overstimulation of these receptors due to either viral or bacterial infections may lead to adverse pregnancy outcomes including preeclampsia (114, 118). Upon trophoblast TLR-3 and TLR-4 activation by microbial byproducts, chemokine secretion initiates the innate immune response and the decidua becomes infiltrated with pNK cells and macrophages (113). TLR4 activation by bacterial LPS, in addition, inhibits trophoblast migration (119), while TLR3 activation by poly I:C, a double-stranded RNA (dsRNA) viral mimetic, increases inflammation and results in the development of preeclampsia-like symptoms in pregnant rats (120). Increased immunoreactivity of the TLR4 protein in placentae from complicated pregnancies suggests that their role in the activation of the innate immune system is in response to the presence of infectious agents (112). It has been recently shown that the expression of TLR4 in placentae from women with EOPE was higher than TLR4 expression in women with LOPE (121). It is possible that this upregulation is part of a compensatory mechanism in preeclampsia, given that higher expression of TLR4 has been described in human term placentae compared to first trimester (122). Activation of TLR3 in pregnant mice increased systolic blood pressure and endothelial damage, both of which were further exacerbated in the absence of IL-10 (123). Moreover, dsRNA and single-stranded RNA (ssRNA) were shown to upregulate expressions of TLR3, TLR7, and TLR8 in mouse placentae. This caused pregnancy-dependent hypertension, endothelial dysfunction, and placental inflammation (124). Women with preeclampsia displayed activation of the aforementioned TLRs; however the association between severity of the disease and activation of TLRs was not confirmed (124). Increased expression of TLR9 in the placentae and peripheral blood mononuclear cells from women with preeclampsia compared to normotensive controls has also been described (125, 126). A study by He et al. showed that when mice were treated with a TLR9 agonist, they developed preeclampsia-like symptoms. This preeclampsia murine model also showed that with exogenous overexpression of TLR9, the levels of sFlt-1 increased while VEGF was downregulated. This suggests that TLR9 is capable of suppressing angiogenesis (127) and that aberrantly activated ligand binding to different TLRs may significantly influence pregnancy outcomes. In a relatively recent study, it was demonstrated that inhibition of TLR activation and thus inhibition of downstream signaling, could not prevent embryo resorption in the absence of dNK cells (105, 128). Differential expressions of TLRs throughout pregnancy and in preeclampsia, suggest that these receptors might represent potential therapeutic targets.

## The Role of Innate B1 Cells in Preeclampsia

As indicated above, there are two different subsets of B1 cells. B1a cells are CD5^+^ and produce “natural antibodies,” which are polyreactive, low-affinity and self-reactive antibodies. On the other hand, B1b cells are CD5^−^ and produce adaptive antibodies when exposed to antigens (58). It is, however, the role of B1a cells that is more closely associated with adverse pregnancy outcomes. Namely, the proportion of B1a cells decreases throughout gestation likely as a protective mechanism against poly-reactive antibodies produced by B1 these cells, which recognize and target a variety of antigens including fetal antigens (129). Their role in preeclampsia has not fully been investigated. However, there are studies emerging regarding their association with hypertensive disorders in pregnancy. The number of peripheral blood B1a cells in women with preeclampsia is significantly increased compared to normal pregnant women (60), however no difference in their number between severe and mild preeclampsia has been observed (130). In addition to the well-established Th1/Th2/Th17-Treg paradigm of the pathogenesis of preeclampsia [as reviewed in (131)], the role of B1 cells is likely linked to stimulation of CD4^+^ T cells and their differentiation into Th17 effector cells (132). It has also been demonstrated that B1a cells can produce agonistic autoantibodies to AT1-AA in pregnancy, which can lead to the development of preeclampsia (133). High affinity binding of AT1-AA to receptors within the placenta leads to increased secretion of anti-angiogenic factors (sFlt-1 and Endoglin), both of which are associated with the onset of preeclampsia (134–136). These autoantibodies appear to correlate with severity of preeclampsia (137). The depletion of B-cells in an animal model of preeclampsia resulted in a decrease in the level of AT1-AA and a reduction in preeclampsia symptoms (138). Natural antibodies secreted by B1a cells are mostly IgM antibodies and are important in clearing and neutralizing pro-inflammatory targets (139). Although the specific roles of B1 cells have not been elucidated yet, their numbers were not significantly increased following placental ischemia (140). Substantial depletion of B cells by the monoclonal anti-human CD20 antibody, rituximab, interestingly did not have a significant effect on the hypertensive response in the RUPP model (140).

In summary, only a limited number of studies have assessed the role of innate B1 cells in preeclampsia. Further research is needed to evaluate the association of innate B1 cells with hypertensive disorders in pregnancy, as well as their role and pathogenic mechanisms in EOPE vs. LOPE.

## Emerging Role of ɤδ T Cells in Preeclampsia

Within the decidua, ɤδ T cells despite originating from the lymphoid lineage facilitate proliferation of trophoblast cells while concurrently suppressing their apoptosis through the secretion of IL-10 (141, 142). This ensures adequate migration and invasion of trophoblast cells leading to appropriate placental development. The role of γδ T cells has not yet been determined in preeclampsia, but increases in the production of pro-inflammatory stimuli, interferon (IFN)-γ & IL-17, by γδ T cells have been reported in women with idiopathic recurrent pregnancy loss (143). Furthermore, in mice, a competitive antagonist binding of the histocompatibility complex (MHC) class II found on the surface of ɤδ T cells, resulted in the reduction of their immunological capabilities (144). The ɤδ T cell “knockout mice” displayed a resistance to developing preeclampsia-like features, implying that these cells could have a role in the pathogenesis of the condition (144). In the same study, preeclamptic placentae demonstrated significantly increased levels of γδ T cells (144).

## The Dysregulation and Over-Activation of the Complement System During Preeclampsia

The distribution and activity of the complement system's components vary between EOPE and LOPE, likely stemming from their different underlying pathogeneses. Dysfunction related to the complement system in EOPE has been correlated with single nucleotide polymorphisms (SNPs) as demonstrated by Wu et al. (145). More specifically, C6 (rs7444800, rs4957381) and MASP1 (rs1108450, rs3774282, rs698106) polymorphisms were shown to correspond independently to a risk of EOPE and severe preeclampsia (145). Another modification to the complement system that is unique to EOPE is the reduction in the placental concentrations of complement 3a receptor (C3aR) mRNA and protein (146). These reductions lead to an increase in the plasma concentration of C3a, the ligand for this receptor (146). Lokki et al. expanded upon these findings and compared the activation of the complement pathways in EOPE vs. normal pregnancies. In their cohort study of 22 women, those with EOPE displayed higher placental deposition of C1q, specifically proximal to areas of fibrinoid necrosis (147). They demonstrated that 43% of EOPE cases had a gene deficiency for C4a/b, a deficiency known to also be implicated in certain autoimmune disorders (147). Finally, Lokki et al. noted that areas of C3b deposition were positively correlated with C1q and negatively with Factor H, a regulatory factor of the alternative pathway (147). The over-activation of the complement system in EOPE is reinforced by the rise in the plasma concentration of C5b-9, which is indicative of terminal activation (148). C-reactive proteins of the system, specifically, C3a, have also been found circulating in high concentration within the amniotic fluid in EOPE (149).

LOPE shares many characteristics with EOPE in dysregulation of the complement system, with some key distinctions. As in EOPE, the MASP1 gene has been shown to display SNPs, however, in LOPE the variants indicated are rs1357134 and rs698090 (145). The aforementioned variations in the genes are completely different from the ones detected in EOPE cases and are specifically correlated with LOPE (145). Examining the sera of both EOPE and LOPE, severe preeclampsia cases revealed some degree of activation of the complement system, as demonstrated by Jia et al. (150). Serum levels of C1q, Factor H, C3 and C4 significantly decreased, while the Bb concentration increased in the presence of either EOPE or LOPE compared to their respective controls (150). Despite this, the concentrations of the C-reactive proteins observed in LOPE were not significantly different than in the EOPE cohort (150). Nevertheless, another recent study by He et al. using similar sample size, characterized the components of the complement system using plasma samples from 30 EOPE and 30 LOPE patients with severe preeclampsia. The results obtained contradicted Jia's investigation, showing elevated Bb, C3a, C5a, and MAC in both EOPE and LOPE, whereas LOPE was specifically associated with elevated C1q and C4d compared to normotensive controls (151). Lokki et al. built upon this data, by inspecting the dissimilarities of C1q deposition in the STB layer of the placenta of LOPE patients. This investigation revealed that the C1q deposits negatively correlated with Factor H, characterizing a shift toward activation within the complement system (147).

## Syncytiotrophoblasts Play Important Role in Preeclampsia

STBs form the feto-maternal placental barrier, which separates the fetal and maternal circulations (65). The STB-containing layer, as described above, is shed into the maternal circulation by the placenta during normal pregnancy, releasing STBMs (152, 153). STBMs levels were increased in EOPE compared to matched normal pregnancies, whereas no change was observed between LOPE and normal pregnancy samples (154). This increase in STMBs potentially contributes to endothelial dysfunction and systemic inflammation (155). Another study confirmed no significant difference between levels of EVs from various cells including STBs, in normal pregnancy compared to LOPE (156). Further studies are needed to determine whether this shedding is potentially more prominent in EOPE compared to LOPE. STBM shedding has been linked to increased levels of active tissue factor, leading to enhanced aggregation of platelets (157). This is evident in EOPE with severe features, but not observed in LOPE, which supports evidence suggesting two distinct phenotypic pathogeneses. Further studies are needed to explore if higher levels of STBMs in EOPE are due to their prevalence being greater in early gestation, independent of the presence of preeclampsia (153). STBMs act as ligands for receptors, growth and coagulation factors, and RNA molecules, and have an important role in cell-cell communication (65). STBMs bind TLRs and activate monocytes, DCs, NK cells, and neutrophils. The subsequent release of various inflammatory cytokines and superoxide radicals contributes to the systemic inflammation associated with preeclampsia (74, 88, 94, 158).

The release of sFlt-1 from STBs exerts indirect anti-angiogenic effects by competitively blocking binding of VEGF and PIGF to their respective receptors (158, 159). Levels of sFlt-1 are increased in preeclampsia and can be used as a biomarker of STB stress associated with EOPE (67, 158). LOPE does not present with this early pathology, with studies reporting changes in angiogenic biomarkers near term, observing similar plasma concentrations in both normal pregnancies and LOPE, thus not providing reliable detection of LOPE (160). Contrary to findings describing the prominent role of STBMs, it has been suggested that soluble factors directly released from STBs mediate endothelial dysfunction in preeclampsia rather than EVs (161).

## MSC Regulation of Innate Immune System Response is Impaired in Preeclampsia

Increased attention has been directed toward investigating the role of MSCs and their immunomodulatory capacity during pregnancy and its complications. As their potential therapeutic role in preeclampsia has been discussed elsewhere (70, 162, 163), here we discuss their contribution to irregular innate immune system signaling in preeclampsia. MSCs are found in many tissues, such as bone marrow, and adipose, decidual and fetal tissue (164–166). Adipose-derived MSCs have demonstrated impaired function associated with senescence in women with preeclampsia (167). Decidual MSCs mediate appropriate placentation and ensure immune tolerance to the semi-allograft fetus (168, 169). These decidual MSCs have the ability to decrease NK cell cytotoxicity and cytokine production (170). This may potentiate the transition of peripheral into decidual NK cells, a process critical for adequate decidual function. Decidual MSCs in addition regulate dNK through their intracellular cytokine expression profile, including TNF-α and IL-4 and via the interaction between collagen and LAIR-1 (171). Bone marrow-derived MSCs are also capable of modulating NK cells by inhibiting their proliferation, cytokine secretion, and cytotoxicity against HLA-class I- expressing targets, either via soluble factors or via cell-to-cell specific interactions (172, 173). A study by Aggarwal and Pittenger showed that immunosuppressive MSC features are associated with the inhibition of TNF-α and IFN-γ, and the secretion of prostaglandin E_2_ (PGE2) (174, 175). Notably, it has been previously suggested that the lack of this prostaglandin in preeclampsia leads to a decrease in both renal blood flow and sodium excretion (176). The immunomodulatory interactions between MSCs and NK cells, along with existing studies, provide promising results that strengthen the potential immunomodulatory effects of MSCs. Although MSC are considered privileged immune cells, they can be recognized and eliminated by activated NK cells (172).

Human placental MSCs also have an immunoregulatory effect on macrophage differentiation, favoring the expression of the M2-immunosuppressive phenotype (177). This immunoregulatory effect may be mediated by soluble molecules acting partially via glucocorticoid and progesterone receptors. MSC treatment decreases IL-6 and TNF-α, while increasing anti-inflammatory cytokine, IL-10 (178). A previous study has suggested that PGE2 plays an important role in the immunoregulatory effects of MSC, indicating that M2 macrophage polarization is initiated via the COX-2-PGE2 pathway (178, 179). MSCs are the most widely used stem cell-based therapies due to their beneficial immunomodulation, anti-oxidant, pro-angiogenic, and regenerative therapeutic effects. Their therapeutic potential for the prevention and treatment of preeclampsia is emerging from a number of pre-clinical studies, which show the ability of MSCs and their associated EVs to abrogate symptoms and features of preeclampsia (reviewed in (70). Their relevance specifically to EOPE and LOPE needs to be elucidated further.

## Therapeutic Strategies for Targeting Innate Immune System Aberrant Mechanisms as Potential Treatments for Preeclampsia

Finding potential novel treatments for preeclampsia is an area of unmet clinical need and is inherently challenging. Significant knowledge gaps exist surrounding the safety, effectiveness and long-term effects of drugs for the use in pregnancy (180). Clinical trials investigating therapeutics that could be potentially repurposed for preeclampsia often have pregnancy as an exclusion criterion because of possible teratogenic risks or other harmful effects to the fetus (Table 1). Consequently, phase 2 or 3 trial data in pregnancy are generally lacking, making it difficult to inform novel treatment strategies. Physiological changes occur in nearly all organs during pregnancy and the pharmacokinetics and pharmacodynamics of drugs are often significantly altered, although the specific changes are mostly undetermined (212). New micro-physiological systems technology such as “Organ on a chip” models may in the future be used to help fill these gaps in knowledge (213).

**Table 1:** Therapeutic strategies targeting aberrant innate immune system mechanisms implicated in preeclampsia.

**Innate immunity target**	**Treatment**	**Mechanism**	**Safety in pregnancy**	**References**
Macrophages	Salidroside (SLDS) is a phenylpropanoid glycoside extracted from the root of *Rhodiola rosea* L	Reduction in M1 macrophage/microglia polarization and an increase in M2 macrophage/microglia polarization in mice	Unknown	(73, 181)
Macrophages	Macrophages transplantation	Increase in M2-polarized macrophages	Risk for fetal and maternal micro-chimerism	(182)
Neutrophils	Maternal corticosteroid administration- Betamethasone	Reversal of delayed neutrophil apoptosis (returning the normal rate of spontaneous neutrophil apoptosis)	Betamethasone acetate Category C (TGA) Betamethasone dipropionate Category B1 (TGA)	(90)
STBM	Neprilysin (NEP) inhibitors Racecadotril (Hidrasec®)	Inhibition of STBM released, promoting vasodilatation, and natriuresis	Category B1 (FASS)	(183, 184)
Maternal microbiome	Probiotic-rich food Milk-based probiotics e.g., *Lactobacillus acidophilus* and *Lactobacillus rhamnosus*	Consumption of probiotic-rich food during pregnancy has been associated with lower rates of preterm birth and preeclampsia Probiotics have been implicated in the modification of placental trophoblast inflammation, systemic inflammation, and blood pressure, all features of preeclampsia *Lactobacillus* could be associated with lower risk of preeclampsia in primiparous women Overstimulation of the innate immune system due to dysbiosis of the maternal microbiome has been linked to preeclampsia	Generally recognized as safe (GRAS) by FDA	(185–188)
IL-10	Recombinant Human Interleukin-10	Increased anti-inflammatory capacity	Recombinant IL-10 reverses hypoxia-induced effects in pregnant mice No significant effect on fetal development in mice	(189–191)
TNFα	Infliximab	TNFα antagonist Anti-inflammatory effects	Category B (FDA) No increases in miscarriage, structural neonatal malformations or prematurity were observed compared with non-exposed pregnancies	(85, 192)
Complement system	Ravulizumab (Ultomiris®)	Inhibit cleavage of C5 into C5a and C5b	Category B2 (FASS)	(193)
TLR9	TLR9 antagonist Low-dose naltrexone (LDN)	Reduced inflammatory activity (studied in Crohn's disease)	Category B3 (FASS)	(194–196)
TLR2 & TLR4	Sparstolonin B (SsnB) derived from the Chinese herb *Spaganium stoloniferum*	Blocks TLR2- and TLR4-mediated NFκB activation in mouse macrophages induced by LPS and Pam3CSK4	Anti-angiogenic and anti-estrogen toxicity effects in pregnant rodents	(197)
TLR4	Ibudilast	Upregulation of anti-inflammatory cytokines (IL-10, IL-4) Antagonism of TLR4	Not tested in pregnant women	(198) www.clinicaltrials.gov (NCT01389193)
TLR9	TLR9 inhibitory oligodinucleotide (ODN2088)	Antagonism of TLR9 associated with reduction in systolic blood pressure	No adverse effects were observed in mice receiving this treatment in a model of type 1 diabetes mellitus ODN2088-treated mice gave birth to healthy pups	(199–201)
TLR4	Berberine- isoquinoline alkaloid mainly extracted from *Rhizoma Coptidis*	LPS antagonist Inhibition of LPS/TLR4 signaling	Berberine can cause or worsen jaundice in newborn infants and could lead to kernicterus	(202–206)
TLR4/NF-κB pathway	Parthenolide- Feverfew (*Tanacetum parthenium* L.)	Inhibition of the TLR4/NF-κB pathway	Not safe in pregnancy Feverfew (Tanacetum parthenium L.) shows potential emmenagogue activity and induces abortion	(202, 207, 208)
IL-1 beta	Canakinumab	Antibody targeting IL-1β Suppression of the innate immune response and systemic anti-inflammatory effects	Category B1 (FASS)	(209)
MSC	MSC-derived EVs	MSC-derived EVs containing molecular cargo and functional mitochondria metabolically reprogram macrophages M1 pro-inflammatory phenotype toward M2 anti-inflammatory phenotype	Unknown/no major adverse effects were reported in preclinical studies with pregnant rodents	(210) (Reviewed in (70)
MSC	PLacental eXpanded (PLX-PAD) cells	Suppress TLR-induced inflammation. Release anti-inflammatory cytokines (IL-15 & GM-CSF) and growth factors (EGF & VEGF-A)	No detrimental effects on fetal development of mice pups	(162)

*The Swedish classification system (Farmaceutiska Specialiteter i Sverige (FASS), American Food and Drug Administration (FDA) and Australian Therapeutic Goods Administration (TGA) were used to determine the safety profile of drugs used during pregnancy. FASS reports on medications on the European market and reflects international text book recommendations (211)*.

*Category A—safe in pregnancy; Category B1, B2, B3—unknown risk in pregnancy or based on animal studies/Categories B (C and D)—unsafe in pregnancy; Category C—possible harmful effects on the human fetus or neonate without causing malformations. The “probably safe” group include FASS and Australian categories A, B1, and B2 and FDA categories A and B; the “potentially risky” group include FASS and Australian categories B3, C, and D, Australian category X, and FDA categories C, D, and X*.

Dysregulation of TLRs and detection of host-derived DAMPs contribute to the pathogenesis of preeclampsia, as described above (214). Novel TLR antagonists, especially inhibitors of TLR4 and TLR9, have potential as exciting new therapeutic agents for inflammatory disorders. The anti-inflammatory properties of TLR antagonists have been explored in numerous clinical trials for diseases such as systemic lupus erythematosus, infection-associated sepsis and vascular disorders such as hypertension (194), yet it is unknown if these agents are safe to use in pregnancy. This is a research area that therefore warrants further investigation perhaps in pre-clinical models of preeclampsia. Many of the aforementioned immune cells have similar unexplored potential and are presented in Table 1. The understanding of the role of the innate immune system in the multifactorial pathogenesis of preeclampsia has been significantly advanced. This progress makes novel therapeutic strategies for targeting aberrant mechanisms within the innate immune system possible as potential treatments for preeclampsia. To support this advancement, greater research capacity and robust and safe clinical trials with pregnant women are needed, with particular focus on delineating differences in EOPE and LOPE management. Anti-inflammatory and immunomodulatory drugs used for other diseases may not be appropriate and safe to use in preeclampsia. It is important to rule out drugs that are not suitable for repurposing in order to streamline future research strategies to focus on more viable alternatives.

## Discussion

There is a plethora of evidence supporting the role of the maternal innate immune system in the pathogenesis of preeclampsia. Mechanisms of irregular signaling and function of the innate immune cells could be explored as potential biomarkers or therapeutic targets in preeclampsia. Moreover, these cells appear to play different roles in the two phenotypes of preeclampsia, EOPE and LOPE, which could lead to better risk stratification and personalized management of preeclampsia. Developing reliable predictive and diagnostic biomarkers especially for LOPE has been challenging given that preeclampsia is a multifactorial disease with a poorly understood pathogenesis (215). As depicted in this review, there are a number of different cell types, both from the innate immune system and other supportive systems such as MSCs and STBs, which if exhibiting irregular signaling, can lead to the development of EOPE or LOPE, or both (Figure 2).

While in some cases quantifying a particular cell types could be utilized as a biomarker of the disease, such as the number of innate B1 cells or NK cells (both pNK and dNK), the mechanisms involved are often diverse and therefore a panel of biomarkers would be necessary to accurately predict or diagnose preeclampsia. Given that the two phenotypes of preeclampsia are often not considered and distinguished in research, it is encouraging that in terms of the innate immune system, there is important evidence emerging regarding the influence of the innate immune system in both EOPE and LOPE. For example, both dNK and pNK cells, as well as TLR4, are likely increased in EOPE, whereas in LOPE there does not appear to be a difference in these factors compared to healthy pregnancy. Another frequently observed difference between EOPE and LOPE is the proliferation of neutrophils and neutrophil associated processes, with increases in NLR, elastase and NETs being much higher in EOPE than in LOPE. Macrophages and innate B1 cells, on the other hand, do not seem to be dysregulated differently between EOPE and LOPE.

Despite the emergence of novel research highlighting the differences in the behavior of certain innate immune cells in terms of the pathogenesis of EOPE and LOPE, it is important to acknowledge that given the complexity of this condition, there are often inter-personal variations in both the mechanisms and symptoms of the disease. Consequently, these factors impose further difficulties in monitoring, diagnosis, and treatment of preeclampsia. In light of this, it is not surprising that there is a lack of effective treatment strategies for this devastating pregnancy condition. A holistic approach to disease monitoring is necessary to identify women at high risk of developing preeclampsia in conjunction with determining a panel of biomarkers representative of the multifactorial nature and different phenotypes of this disease.

Our evaluation of the existing literature describing interactions between maternal innate immune cells and cells of placental/uterine origin identified a number of limitations in the field. As the heterogeneous nature of preeclampsia has only been recently classified, there is a delay in current research, with a limited number of studies fully examining the interplay among innate immune cells and components of the placenta in the context of both EOPE and LOPE. Certain cell types, nevertheless, have been well-characterized for these two phenotypes of preeclampsia. Evidence is lacking, however, for other cell types of the innate immune system regarding their involvement in the pathogenesis of preeclampsia regardless of the phenotype. These include eosinophils, basophils, mast cells, DCs, and Langerhans cells. It is possible that some of these do not play an important role in the development of preeclampsia. However, given the key roles of mast cells and DCs in pregnancy (Figure 1) and some evidence of their roles in the placental bed in preeclampsia, albeit with conflicting results (40), it is likely that these cells could influence preeclampsia monitoring and treatment in the future. A portion of the reviewed literature did examine the various cell types discussed above, however, evidence was provided regarding their behavior in cases of mild and severe preeclampsia, rather than EOPE and LOPE. As a consequence, while there is currently some literature reporting on the behavior of innate immune cell types in preeclampsia, more substantial evidence is required to accurately distinguish immune cell behaviors in both phenotypes of the condition.

Carrying out research with vulnerable groups such as pregnant women is inherently challenging and results in certain limitations. Adherence to stringent ethical considerations and difficulty in obtaining early placental tissue reduces the ability of an investigation to fully elucidate the roles that the immune cells may play in the pathogenesis of preeclampsia. Recent developments in a number of microfluidics or 3D multicellular platforms may greatly increase our understanding of the cellular and molecular mechanisms of the innate immune system associated with inadequate remodeling of spiral uterine arteries or placental development/growth relevant to preeclampsia. The DAX-1™ chip manufactured by AIM Biotechnology has been demonstrated to successfully and accurately recapitulate human tumor immune microenvironments (216). Utilizing this microfluidics platform, investigators were able to examine cell type dependent interactions and provide a novel insight into the tumor immune responses (216). Utilization of these or similar platforms might be able to reproduce the multicellular autocrine and paracrine conditions of preeclampsia, and the behavior of innate immune cells within the microenvironment could be further studied. Thus, researchers can circumnavigate the hurdles of collecting early pregnancy placental tissue while still producing accurate and relevant data. Replicating an EOPE or LOPE environment will be challenging given the distinct and overlapping features of these two phenotypes of preeclampsia. Nevertheless, additional benefits of the microfluidics platforms include the ability to track molecular changes in real-time and the potential to test emerging drug treatments.

## Conclusion

Components of the innate immune system are fully or partially inactivated, or experience a tolerogenic shift in their immunological function throughout gestation. This, in conjunction with the ability of certain placental cells to modulate the immune system, confers a level of protection to the developing fetus against detrimental immunological responses. This delicate balance is disrupted in preeclampsia, leading to the inappropriate over-activation of these immune cells, causing disruption of appropriate placentation and contributing to the development of this hypertensive condition with end-organ damage. Although the dysfunction of these cells is observed in LOPE, the imbalance appears to be most pronounced in EOPE. While existing literature provides some evidence regarding the roles of the innate immune cells, including NK cells and neutrophils in EOPE and LOPE, further investigation specifically in the context of both phenotypes of preeclampsia, is required to address knowledge gaps. This could lead to the identification of specific disease mechanisms, which could be explored as new diagnostic biomarkers or treatment targets, hence improving the management of preeclampsia and identifying potential emerging treatments for both EOPE and LOPE.

## Author Contributions

IA, SS, and DP carried out literature search and created a draft of the manuscript. LM, VG, and TS conceptualized the topic, supervised, and revised the draft. All authors approved the manuscript.

## Conflict of Interest

The authors declare that the research was conducted in the absence of any commercial or financial relationships that could be construed as a potential conflict of interest.
